# Partial Characterization of Protease Inhibitors of *Ulva ohnoi* and Their Effect on Digestive Proteases of Marine Fish

**DOI:** 10.3390/md18060319

**Published:** 2020-06-18

**Authors:** Antonio Jesús Vizcaíno, Alba Galafat, María Isabel Sáez, Tomás Francisco Martínez, Francisco Javier Alarcón

**Affiliations:** Departamento de Biología y Geología, Escuela Superior de Ingeniería, Ceimar-Universidad de Almería. La Cañada de San Urbano, 04120 Almería, Spain; albagalafat@gmail.com (A.G.); msc880@ual.es (M.I.S.); tomas@ual.es (T.F.M.); falarcon@ual.es (F.J.A.)

**Keywords:** inhibitor, macroalgae, marine fish, protease, *Ulva ohnoi*

## Abstract

This piece of research evaluates the presence of protease inhibitors in the macroalga *Ulva ohnoi* and provides an initial overview of their mode of action. The ability of *Ulva* protease inhibitors to inhibit digestive proteases of three marine fish species, as well as their capacity to hamper the hydrolysis of a reference protein by those fish proteases, were assessed. In addition, thermal stability and the mode of inhibition on trypsin and chymotrypsin were also studied. Dose-response inhibition curves and in vitro protein hydrolysis assays revealed a noticeable inhibition of fish enzymes when *Ulva* concentration increased in the assay. The thermal treatment of *Ulva* reduced markedly the inhibitory effect on fish digestive protease. Finally, Lineweaver–Burk plots indicated that trypsin and chymotrypsin inhibition consisted of a mixed-type inhibition mechanism in which the inhibitory effect depends on *Ulva* concentration. Overall, the results confirmed the presence of protease inhibitors in *Ulva,* though heat treatment was enough for inactivating these compounds.

## 1. Introduction

Anti-nutritional factors (ANFs) can be defined as substances that, by themselves or through their metabolic products, can exert negative effects on food utilization and interfere with the normal growth, reproduction and health of fish [1]. From a nutritional point of view, the presence of these compounds in diets is responsible for the deleterious effects on the absorption of nutrients and micronutrients, which may interfere with the normal functioning of certain organs [2]. This fact is one of the most important issues derived from using novel dietary ingredients in aquaculture, as well as one of the main drawbacks that limits their practical application in formulated feeds [3,4].

In general, ANFs have been mainly related to plant-derived feedstuffs, and they comprise a wide variety of compounds, such as protease inhibitors, phytohemagglutinin, lectins, phytic acid, saponins, phytoestrogens or antivitamins [1,5]. Although less known, recent studies have also documented the presence of these substances in seaweeds, considered currently a potential alternative ingredient for farmed fish [6,7].

Seaweeds have drawn the attention of researchers not only as an important source of dietary protein, but also as functional ingredients in aquafeeds [8]. More specially, some species of the genus *Ulva* have been successfully evaluated as a dietary ingredient in some farmed fish species, such as gilthead seabream (*Sparus aurata*) [9,10], Senegalese sole (*Solea senegalensis*) [8,11] or seabass (*Dicentrarchus labrax*) [12], with promising results in terms of growth, survival and nutrient utilization. Despite the health benefits reported for seaweeds, some studies have described that the dietary inclusion level of algae above 20% yields detrimental effects on fish growth and other zootechnical parameters. It has been suggested that these effects could be attributed to the existence of ANFs, which might affect the bioavailability and/or digestibility of nutrients [13]. 

In this regard, Oliveira et al. [14] and Maehre, [6] confirmed the presence of lectins, trypsin and alpha-amylase inhibitors, as well as phytic acid, in some species of marine algae, although polyphenolic compounds are considered the substances most frequently linked to their antinutritional effects [6,14]. It has been described that the presence of lectins in feed alters the intestinal epithelium, resulting in the over-secretion of mucus that may impair the enzymatic and absorptive capacity of fish [15], altogether leading to reduced growth [5]. Phytic acid and polyphenols bind to proteins and polysaccharides producing insoluble high-molecular complexes, a fact that reduces nutrient bioavailability and consequent nutrient deficiency, such as that described for methionine, which is essential for lipid metabolism [15,16]. In addition, other antinutritional compounds of seaweeds, such as phytates, can inhibit the action of gastrointestinal enzymes like tyrosinase, trypsin, pepsin, lipase and amylase [2].

It is also worth mentioning the existence of protease inhibitors, substances that bind to proteolytic enzymes causing not only reduced proteolysis, but also increased pancreatic secretion as an attempt to overcome these antinutritional effects [17]. Despite the evidence suggesting the presence of protease inhibitors in seaweeds and their possible effects on the digestive physiology of aquacultured fish [7,13,17], scarce research is available regarding the characterization of their effects. In this context, this this research aims to assess the presence of protease inhibitors in *Ulva ohnoi*, evaluating the effects of such inhibitors on fish digestive proteases, characterizing their mode of inhibition, and exploring potential strategies to reduce their detrimental effects on fish digestive enzymes. 

## 2. Results

### 2.1. Inhibition Assay

**Figure 1:**
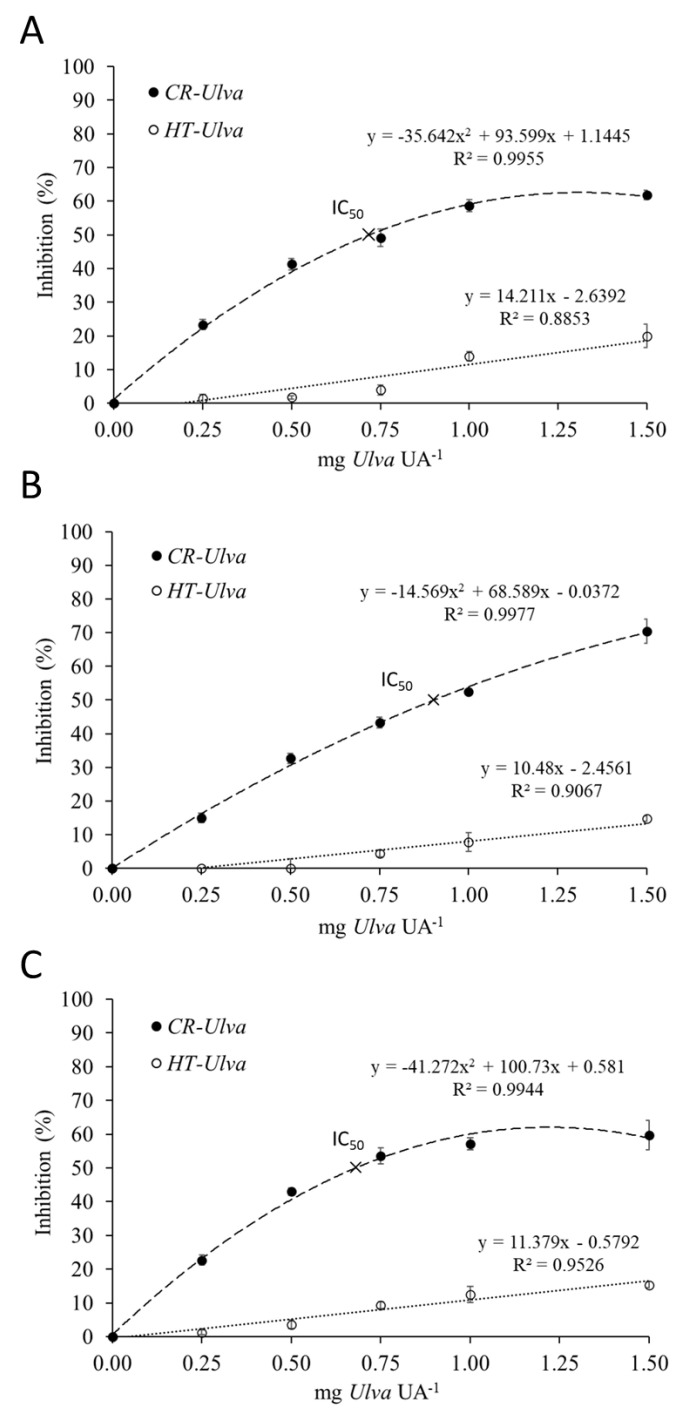
Dose-response inhibition plot of *S. aurata* (**A**), *S. senegalensis* (**B**) and *D. labrax* (**C**) intestinal proteases activity following the incubation with increasing concentrations of crude (CR-*Ulva*) and heat-treated *Ulva* extracts (HT-*Ulva*). Each point represents the mean of triplicates ± SD. IC_50_: mg of *Ulva* needed to cause 50% protease inhibition.

The inhibitory effect of *Ulva ohnoi* on the digestive proteases of gilthead seabream, Senegalese sole and seabass is shown in Figure 1. Dose-response inhibition curves were obtained by measuring the reduction in proteolytic activity on a standardized fish intestinal extract following incubation with different volumes of *Ulva* extract. The results confirmed the presence of protease inhibitor in crude *Ulva* (CR-*Ulva*) able to inhibit up to 70% of Senegalese sole’s proteolytic activity, and by 65% of protease activity in the other two fish species. It was also found that the amount of *Ulva* able to cause the inhibition of 50% digestive protease activity (IC_50_) ranged from 0.6 to 0.9 mg *Ulva* per unit of proteolytic activity (UA). On the other hand, autoclaved *Ulva* (heat treated; HT-*Ulva*) reduced significantly its inhibitory capacity (less than 20% inhibition in all cases) compared to CR-*Ulva* (*p* < 0.05).

**Figure 2:**
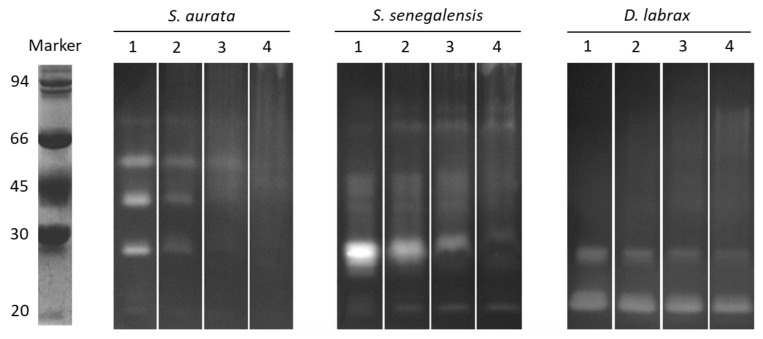
Substrate-SDS-PAGE (sodium dodecyl sulphate polyacrylamide gel electrophoresis) obtained after incubation of *S. aurata*, *S. senegalensis* and *D. labrax* intestinal extracts with different concentrations of *Ulva* extract. Lane 1: control without inhibitor (distilled water was used instead of *Ulva* extract); lane 2: 500 µg *Ulva* per unit of proteolytic activity (UA); lane 3: 1000 µg *Ulva* per UA; lane 4: 1500 µg *Ulva* per UA. The molecular mass markers (kDa) were phosphorylase b (94), bovine serum albumin (66), ovalbumin (45), carbonic anhydrase (30), and soybean trypsin inhibitor (20).

Zymograms of fish digestive proteases after incubation with *Ulva* extract are shown in Figure 2. The effects of protease inhibitors were compared to a control without *Ulva* (lane 1). Noticeable reduction in the intensity of the active fractions (with proteolytic activity) was evidenced after incubation with *Ulva* (lanes 2 to 4). Differences in the inhibitory effect were observed among the three fish species. In the case of gilthead seabream, a progressive decrease in the intensity of all active proteases was observed as the *Ulva* concentration increased, whereas protease inhibition in the other fish was selective for some specific active fractions.

### 2.2. In Vitro Casein Hydrolysis

**Figure 3:**
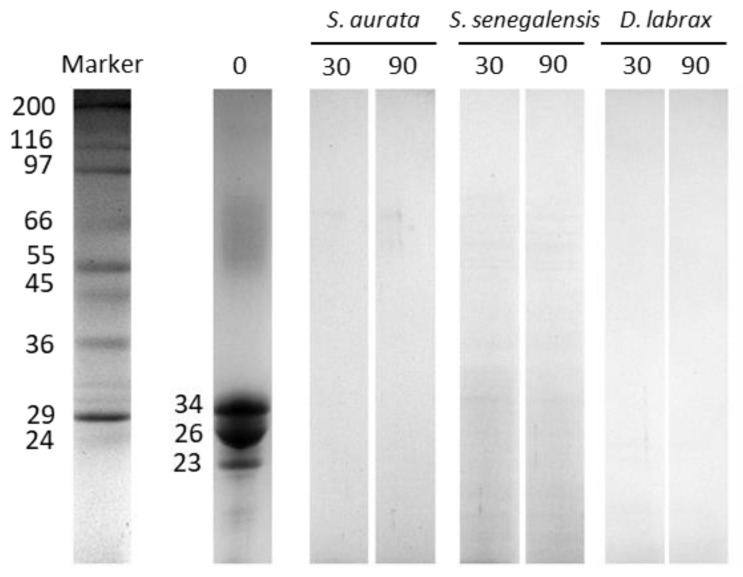
The time-course of in vitro proteolysis of casein by *S. aurata*, *S. senegalensis* and *D. labrax* digestive proteases in the absence of any inhibitory extract. Images show SDS-PAGE hydrolysis patterns obtained at different sampling times (0, 30, and 90 min). Numbers on the left of the electrophoresis gel stand for the molecular mass of proteins fractions (kDa).

Proteinograms of casein hydrolyzed by fish digestive enzymes are shown in Figure 3. All the main casein fractions (34, 26 and 23 kDa), corresponding to α, β and κ subunits, were hydrolyzed after 30 min. 

**Figure 4:**
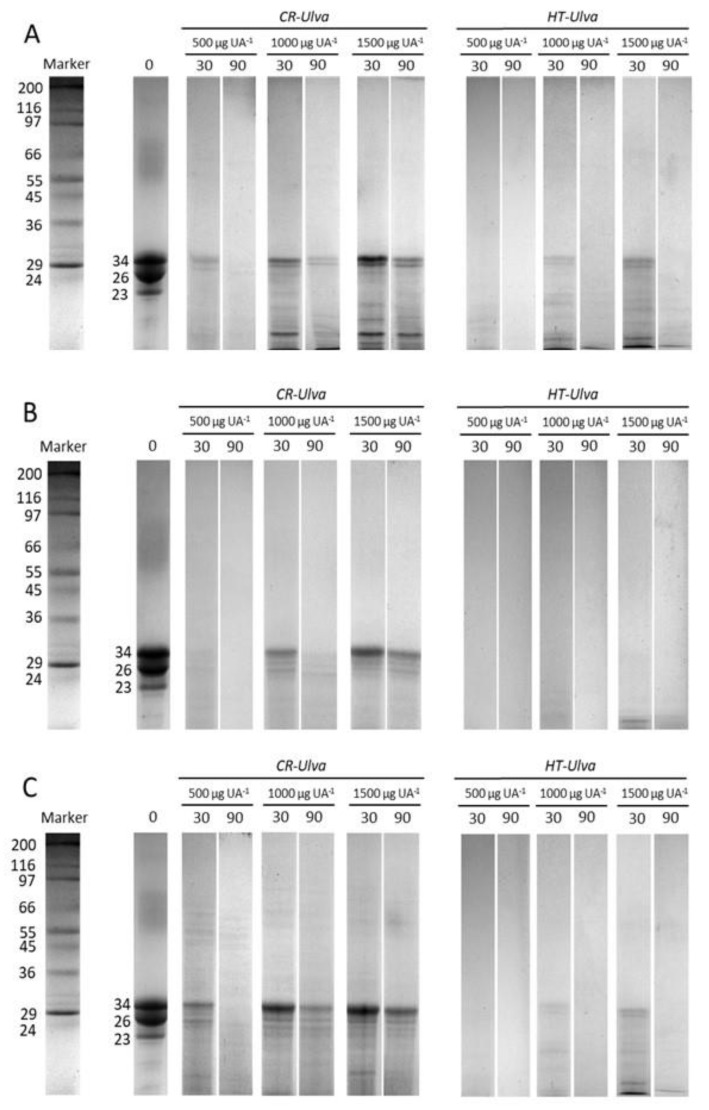
The time-course of in vitro proteolysis of casein by *S. aurata* (**A**), *S. senegalensis* (**B**) and *D. labrax* (**C**) digestive proteases in the presence of increasing concentrations of crude (CR-*Ulva*) and heat-treated (HT-*Ulva*) *Ulva*. The images show SDS-PAGE hydrolysis patterns obtained at different sampling times (0, 30, and 90 min). The numbers on the left of the electrophoresis gels stand for the molecular mass of the protein fractions (kDa).

Figure 4 shows the time-course protein hydrolysis of casein by the fish digestive proteases in the presence of *Ulva* extracts. Overall, CR-*Ulva* hampered the capacity of fish proteases to hydrolyze casein. The fate of the casein fractions throughout the in vitro assay was partial and less marked compared to the same assay carried out without *Ulva* (Figure 3). It was also observed that the inhibitory effect was dose-dependent, given that the higher CR-*Ulva* concentration in the in vitro assay, the lower the hydrolytic capacity of fish digestive enzymes against casein. The inhibitory effect of *Ulva* extracts on digestive proteases was reduced remarkably owing to thermal treatment, as revealed by the proteinograms shown in Figure 4 (lanes at the right). Thus, heat-treated seaweed extracts (HT-*Ulva*) were affected casein hydrolysis to a lesser extent than untreated *Ulva* (CR-*Ulva*), in such a way that none of the casein subunits remained after 90 min in any of the assays carried out with HT-*Ulva*. However, intraspecific and dose-dependent differences were found, and thus increasing concentrations of HT-*Ulva* also reduced casein hydrolysis, not least for seabream and seabass digestive proteases. 

The estimated values of the coefficient of protein degradation (CPD) and total amino acids released after the in vitro hydrolysis of casein are given in Table 1. The CR-*Ulva* lowered the rate of protein degradation compared to the assay performed without *Ulva* (*p* < 0.05). The lowest CPD values were observed for the highest concentrations of CR-*Ulva* in the reaction mixture (1500 µg per UA). In parallel, the amount of free amino acids released was also affected by the interaction of *Ulva* extracts with fish proteases, especially for highest concentrations of CR-Ulva. The impact of HT-*Ulva* on *S. aurata* and *S. senegalensis* digestive protease activity was negligible, since neither the CPD nor free amino acids that were released were different from the controls. However, a certain residual inhibitory effect of HT-*Ulva* was observed on *D. labrax* enzyme extracts. 

### 2.3. Thermal Stability of Protease Inhibitors

**Figure 5:**
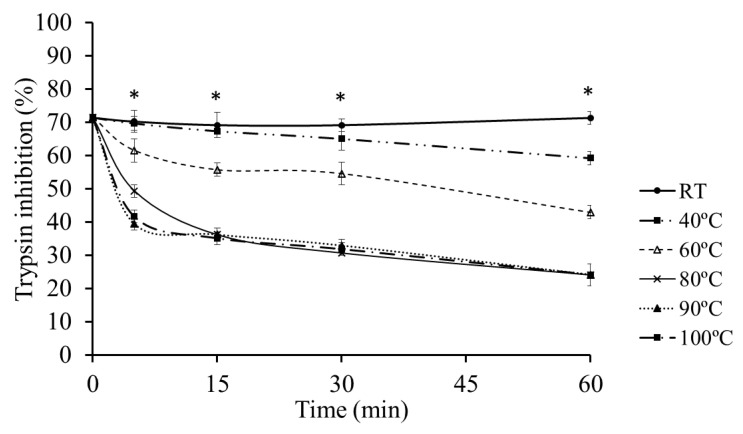
The effects of thermal treatments on the inhibitory effect of *Ulva* on trypsin activity. * Denote significant differences among treatments (*p* < 0.05).

The influence of a thermal treatment on the inhibitory capacity of *Ulva* on trypsin activity was evaluated. A noticeable reduction in the inhibitory effects of *Ulva* was observed as the temperature increased (Figure 5). Protease inhibitors of the *Ulva* extracts were stable at room temperature, but their inhibitory effect significantly reduced (*p* < 0.05) above 40 °C. The exposure of *Ulva* extract at 80 °C for 30 min reduced their inhibitory activity up to 50% compared to the untreated controls.

**Figure 6:**
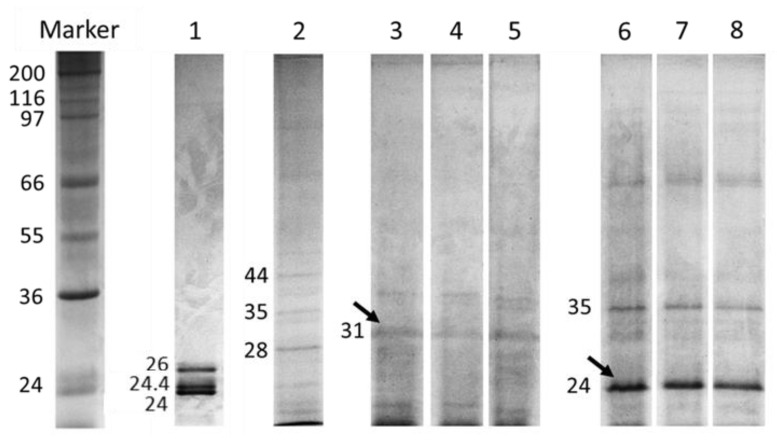
SDS-PAGE carried out to detect trypsin-*Ulva* inhibitor complexes. Lane 1 = trypsin (1 mg mL^−1^). Lane 2 = *Ulva* (0.1 g mL^−1^). Lanes 3 to 5 = trypsin preincubated with CR-*Ulva* during 0, 30 and 60 min. Lanes 6 to 8 = trypsin pre-incubated with heat-treated *Ulva* during 0, 30 and 60 min.

The existence of proteinaceous complexes consisting of *Ulva* protease inhibitors and trypsin is revealed in Figure 6. The commercial trypsin showed three protein fractions (24, 24.4, and 26 kDa, lane 1), whereas the protein pattern of the *Ulva* extract contained several protein fractions (lane 2). The incubation of trypsin with crude *Ulva* before SDS-PAGE (sodium dodecyl sulphate polyacrylamide gel electrophoresis) yielded a characteristic protein profile in which the 24 and 26 kDa fractions of trypsin disappeared. Instead, a new 31 kDa fraction appeared (lanes 3 to 5), which was not present either in trypsin or in *Ulva*. However, this 31 kDa protein fraction did not appear when trypsin was incubated with heat-treated *Ulva* (lanes 6 to 8).

### 2.4. Kinetic Parameter’s

**Figure 7:**
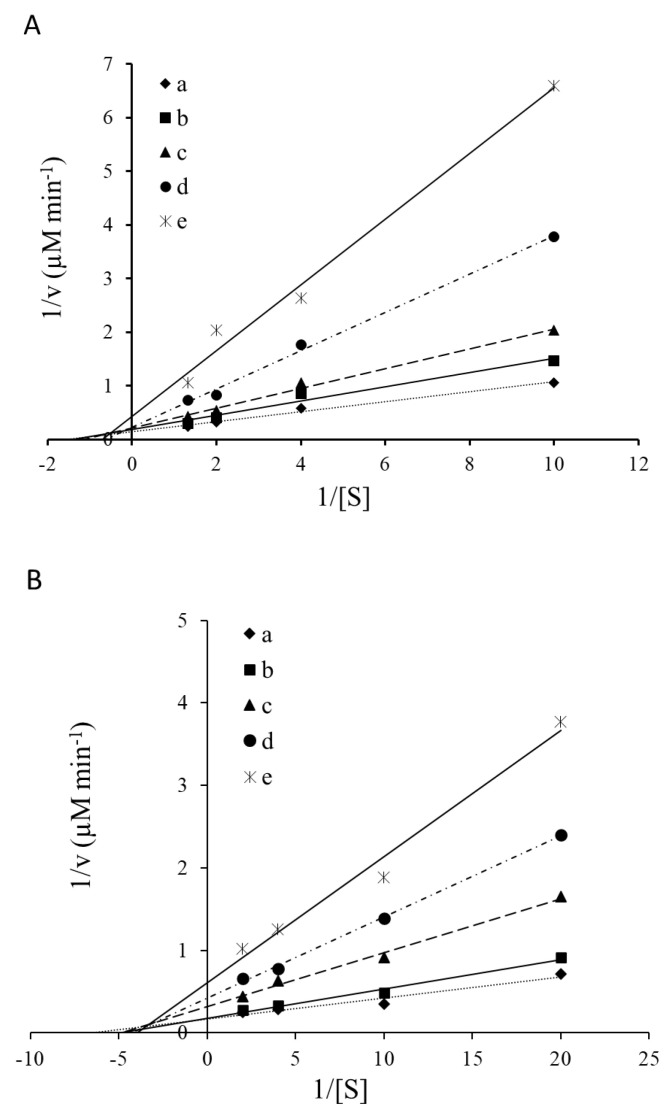
Lineweaver–Burk plots showing the inhibition of trypsin (**A**) and chymotrypsin (**B**) by *Ulva* protease inhibitors (a: 0; b: 200; c: 300; d: 400 and e: 500 µg *Ulva* per µg enzyme).

The data obtained for the kinetic studies performed with trypsin and chymotrypsin are shown in Figure 7. The results demonstrated that *Ulva* protease inhibitors yielded a potential mixed type inhibition, as revealed by the decrease in V_max_ as K_m_ increased, compared to the apparent kinetic parameters obtained for both trypsin and chymotrypsin activities in the absence of *Ulva* (Table 2).

## 3. Discussion

Seaweeds are considered a promising alternative ingredient for aquafeeds. In addition to having nutrients with potential quantitative interest, the presence of biologically active compounds like polysaccharides, pigments (chlorophylls and carotenoids), sterols, polyphenols, and vitamins also makes seaweeds a valuable functional ingredient for aquafeeds [18,19]. However, it has been reported that macroalgae contain several anti-nutritional factors as well, such as lectins and protease inhibitors that might interfere with digestive processes [8]. In this regard, the present study explores the presence of antinutritional factors in *Ulva ohnoi* with a potential inhibitory effect on fish digestive proteases and provides an initial overview of their mode of action on those proteolytic enzymes.

Related to *Ulva* ANFs, the results of the present study confirmed the existence of compounds able to reduce the digestive proteolytic activity of different marine fish species. This fact agrees with previous studies pointing to the existence of protease inhibitors in some macroalgae species, such as *Ulva rigida*, *Ulva ohnoi, Gracilaria cornea* and *Sargassum* sp. [7,8,17]. In our study, inhibition plots and zymograms illustrate the response of fish proteases after incubation with crude *Ulva ohnoi*. Seabream, Senegalese sole and seabass digestive proteases showed susceptibility to *Ulva* ANFs, although a high concentration of *Ulva* was needed to cause high inhibition (>50% of protease inhibition). According to the equations obtained from the inhibition assays, the amount of *Ulva* required to reach IC_50_ would represent a dietary inclusion of approximately 40–53% in feeds, which are unusual levels for feed formulation. From a practical point of view, for 40 g fish consuming feeds containing 15% *Ulva* at an intake rate of 3% of its body weight, a 20% reduction in their digestive protease activity would be expected. In general, fish have physiological mechanisms aimed at compensating the effects of dietary antinutrients [20], although the influence of these compounds is species-specific [21] and may depend on different factors like fish physiology, macroalgae species, duration of feeding period with seaweed-supplemented diets, and the dietary inclusion level [8]. In this regard, there are numerous studies reporting the utilization of dietary seaweeds without compromising growth performance [19,22]. However, others described some negative effects on fish growth, even using seaweed biomass at low dietary inclusion level [8].

The effect of *Ulva* inhibitors on fish digestive proteases were also evidenced in zymograms. Protease inhibition caused by CR-*Ulva* on gilthead seabream enzymes could be classified as “unspecific”, owing to the fact that all the protease fractions visualized in the gels were affected similarly. However, the inhibitory effect seemed to be more “specific” in Senegalese sole and seabass, taking into account that the active fractions with molecular masses below 30 kDa were inhibited even with the lowest amount of crude *Ulva*, whereas heavier proteases were inhibited only using the highest concentration assayed. This reduction in protease activity may negatively affect feed intake and nutrient digestibility in fish [23]. In this context, the in vitro digestive simulations also confirmed that CR-*Ulva* hampered the hydrolysis of standard (casein) by digestive proteases of three different species of aquaculture fish. Thus, a clear reduction both in CPD values and the amount of amino acids released was evidenced when the concentration of CR-*Ulva* in the in vitro assay was increased. Considered together, both findings clearly indicate lower protein hydrolysis and also reflect a significant reduction in the hydrolytic action of both the digestive endo- and exo-proteases of fish [24].

The deactivation of ANFs is an important issue in raw materials processing [25]. Basically, ANFs can be divided into two groups: (i) heat-labile ANFs, including protease inhibitors, phytates and lectins; and (ii) heat-stable ANFs, represented by saponins, non-starch polysaccharides and some phenolic compounds [2]. Heat treatment is a simple procedure for inactivating ANFs and improving the nutritional value of raw protein feedstuffs [26]. The results in our study indicate that thermal treatment is effective when it comes to inactivating *Ulva ohnoi* ANFs that affect fish digestive proteases. The degree of ANFs inactivation depends on factors like temperature, time, particle size, and moisture conditions [2]. In fact, both time and temperature should be controlled carefully in order to minimize losses of nutritional value of a given feed ingredient (for instance, lower availability of amino acids and vitamins, and reduced protein bioaccessibility) as a result of excessive heat denaturation [15,27]. In this regard, the effect of the thermal treatment on the capacity of *Ulva* to inhibit trypsin activity indicates that such inhibitors are susceptible to relatively slight thermal treatment. A thermal treatment of 80 °C for 15 min reduced the inhibitory capacity by 50%, and above 75% as prolonged times were applied. In agreement, proteinograms (Figure 6, lanes 6 to 8) also confirmed that *Ulva* protease inhibitors are thermolabile, owing to the lack of detection of the 31 kDa proteinaceous complex following the thermal treatment of *Ulva*. These results suggest that temperatures reached during the standard industrial processing, for instance, in the extrusion of feeds, would be enough for minimizing the inhibitory capacity of *Ulva*. Both the preconditioning of the ingredient mixture and the friction forces of the extrusion process itself, which squeezed through a cylinder by a specially designed volute [26] can increase the temperature above 100 °C. Indeed, many researchers have shown that extrusion is an efficient procedure for decreasing the trypsin inhibitory capacity of pulses like soybean without altering the amino acid composition, transforming soybean into a high-quality product [26].

For a better understanding of the potential mode of action of *Ulva* protease inhibitors, a kinetic study was performed in which commercial trypsin and chymotrypsin were exposed to *Ulva* extracts. Plant protease inhibitors are characterized by either reversible or irreversible mechanisms [28]. In the present study, kinetics studies revealed a potential reversible inhibition. According to the Lineweaver–Burk plots, both trypsin and chymotrypsin inactivation occurred by a mixed-type inhibition. This type of inhibition is characterized by the ability to bind not only to free enzymes but also to with the enzyme-substrate complexes [29]. In kinetics terms, mixed type inhibition causes changes that result in a progressive decrease in V_max_ when K_m_ increases occurs [30]. This type of inhibition cannot be reversed by increasing substrate concentration, given that the inhibitor cannot be displaced by the substrate. Therefore, the extent of the inhibition depends on the concentration of the inhibitor. In addition, differences in K_m_ indicated that *Ulva* protease inhibitors presented higher affinity for trypsin than for chymotrypsin.

Although plenty of literature regarding the mechanisms of plant protease inhibitors is available, there are no studies assessing the mode of action of seaweed protease inhibitors. Protease inhibitors in soybean and other seeds have been studied extensively. They are grouped into the Bowman–Birk and Kunitz families according to primary structure homology, the position of reactive sites, the number or location of disulfide bonds, and their ability to withstand thermal and acid processing [31]. According to the available literature, Kunitz proteinase inhibitors are usually 18–26 kDa proteins [32]. Overall, they are characterized by several Kunitz domains composed of approximately 60 amino acid residues, stabilized by three conserved disulfide bonds. This family mainly inhibits trypsin and weakly inhibits chymotrypsin [33] and is relatively heat- and acid-sensitive [15]. On the other hand, Bowman–Birk proteinase inhibitors (BBIs) are usually 6–9 kDa proteins with a polypeptide chain bridged by seven conserved disulfide bonds; they have independent sites for trypsin and for chymotrypsin, and they display similar inhibitory capacity for both proteases [31,34]. Disulfide bonds are fundamental for maintaining the structural stability of inhibitors [35]. Unfortunately, this work does not provide information on the structure of purified protease inhibitors of *Ulva*, their molecular weight, or their amino acid profile; however, the ability to inhibit mainly trypsin and also chymotrypsin, as well as the heat lability observed, suggest a certain similarity with the Kunitz type inhibitors family, although further studies are needed to ascertain this hypothesis.

## 4. Materials and Methods

### 4.1. Ulva Biomass

*Ulva ohnoi* biomass was ground, sieved (<100 µm), and kept at −20 °C until use. For inhibitory assays, *Ulva* biomass was separated in two different batches (100 g each), the first batch received no thermal treatment (CR-*Ulva*), and the second was heat-treated at 120 °C for 20 min (HT-*Ulva*). Aqueous extracts (0.1 g mL^−1^) were prepared from CR-*Ulva* and HT-*Ulva*, homogenized in distilled water by shaking for 30 min at room temperature, and then for 24 h at 4 °C. The mixture was centrifuged for 20 min at 12,000 g and 4 °C. Supernatants were stored at 4 °C until used in posterior inhibitory assays.

### 4.2. Fish Enzyme Extracts

Juvenile specimens of gilthead seabream (*Sparus aurata*), seabass (*Dicentrarchus labrax*), and Senegalese sole (*Solea senegalensis*) were used as model aquaculture fish species. Nine fish of each species were anesthetized and sacrificed by severing their spine according to the requirements of the Council Directive 2010/63/UE. The abdomen was opened and the whole viscera were obtained. Intestines of each species were pooled (three pools including three intestines each, one per fish species), and manually homogenized in distilled water at 4 °C to a final concentration of 0.5 g mL^−1^. Supernatants were obtained after centrifugation (12,000 rpm for 12 min at 4 °C) and stored at −20 °C until further use. The total soluble protein in the enzyme extracts was determined using bovine serum albumin as standard [36]. The total alkaline protease activity in the enzyme extracts was measured spectrophotometrically following the procedures described by Alarcón et al. [37], using 5 g L^−1^ casein in 50 mM Tris–HCl (pH 9.0) as substrate. One unit of total protease activity of activity (UA) was defined as the amount of enzyme that released 1 µg of tyrosine per min in the reaction mixture, considering an extinction coefficient for tyrosine of 0.008 µg^−1^ mL^−1^ cm^−1^, measured spectrophotometrically at 280 nm.

### 4.3. Testing the Presence of Protease Inhibitors in Ulva

The inhibitory effects of CR-*Ulva* and HT-*Ulva* on the intestinal proteases of gilthead seabream, Senegalese sole and seabass were determined using a modification of the method described by Alarcón et al. [38]. This method is based on the measurement of the residual proteolytic activity after the preincubation of fish extracts with different volumes of CR-*Ulva* and HT-*Ulva* extracts providing a ratio mg *Ulva* per fish protease activity ranged from 0.0 mg *Ulva* UA^−1^ to 1.5 mg *Ulva* UA^−1^. Enzyme inhibition was expressed as a percentage of protease inhibition after comparing with a control assay carried out without any *Ulva* extract. In addition, the amount of *Ulva* requested for 50% protease inhibition (IC_50_) was estimated. 

In order to visualize the effect of *Ulva* on active fish intestinal proteases, substrate-SDS-PAGE electrophoresis gels were performed. Intestinal extracts were preincubated for 60 min with different volumes of CR-*Ulva* or HT-*Ulva* extracts. Then, the samples were mixed (1:1) with SDS sample buffer (0.125 M Tris HCl, pH 6.8; 4% (*w*/*v*) SDS; 20% (*v*/*v*) glycerol; 0.04% (*w*/*v*) bromophenol blue) and SDS-PAGE was performed according to Laemmli, [39] using 11% polyacrylamide gels (100 V per gel, 45 min at 4 °C). Zymograms revealing protease active bands were made according to Alarcón et al. [37]. After electrophoresis, gels were washed with distilled water and incubated in 0.75% (*w*/*v*) casein solution prepared in 50 mM Tris–HCl buffer, pH 9.0, for 30 min at 4 °C. The gels were then incubated in the same solution for 90 min at 37 °C without agitation. Finally, the gels were washed and fixed in 12% TCA for 10 min to stop the reaction prior to staining with Coomassie Brilliant Blue R-250 in a solution of methanol–acetic acid–water for 12 h. Distaining was done using a methanol–acetic acid–water solution. Clear gel zones revealed the presence of active proteases with caseinolytic activity.

### 4.4. Effect of Ulva on Fish Digestive Proteases

The capacity of *Ulva* to inhibit the hydrolysis of casein by fish intestinal proteases was also assessed using an in vitro assay in the presence of different concentrations of crude (CR) and heat-treated (HT) algae biomass providing 0.5, 1.0 and 1.5 mg *Ulva* UA^−1^.

The in vitro casein hydrolysis was simulated in 10 mL-jacketed reaction vessels connected to a circulating water bath at 37 °C, under continuous agitation by a magnetic stirrer. The temperature was selected in order to increase the activity of the enzymes for reducing the time requested for each analysis [40]. An amount of casein, providing 80 mg of crude protein per vessel, was suspended in 50 mM Tris HCl buffer pH 9.0. After 15 min stirring, the hydrolysis was started by the addition of the enzymatic extract providing 200 UA of total alkaline proteolytic activity [24]. The alkaline hydrolysis was maintained for 90 min, and samples of the reaction mixture (0, 15, 30, 60 and 90 min) were withdrawn. The products of the hydrolysis were separated by sodium dodecyl sulphate polyacrylamide gel electrophoresis (SDS-PAGE), and total amino acids released were also measured at each sampling time, in order to estimate the sequential degradation of casein [41]. All determinations were performed in triplicate. Blank assays with casein but without *Ulva* biomass were carried out for each fish species. 

In order to visualize the casein hydrolysis of SDS-PAGE-separated casein fractions, electrophoresis gels were performed. The procedure was carried out as previously described. The rate of hydrolysis was expressed by a numerical value obtained considering both the percentage of reduction in optical density for each protein after the enzymatic hydrolysis and the relative proportion that such protein represented in the total proteins [38]. The value obtained was called the coefficient of protein degradation (CPD), and it was estimated using the following mathematical expression:

$$CDP=\overset{n}{\underset{i=1}{\sum }}\left[\frac{OD_{i}\left(t=0\right)-OD_{i}\left(t=90\min\right)}{OD_{i}\left(t=0\right)}\;x\;100\right]\;x\;\frac{OD_{i}\left(t=0\right)}{\sum_{i=1}^{n}OD_{i}\left(t=0\right)}$$

where *i* are the proteins identified, *OD_i_* is the optical density of the proteins, and *t* is the time of reaction. 

In addition, the total released amino acids in each sampling time were also quantified at 340 nm in a spectrophotometer (Shimadzu UV-1800, Shimadzu, Kyoto, Japan), using L-leucine as standard [41]. The results were expressed as accumulated values of amino acid released during the enzymatic hydrolysis (g 100 g protein^−1^).

### 4.5. Partial Characterization of Ulva Protease Inhibitors

#### 4.5.1. Effect of Temperature on Protease Inhibitors

The effect of temperature on *Ulva* protease inhibitors was assessed by heating the aqueous *Ulva* extract (0.1 g mL^−1^) at different temperatures (25, 40, 60, 80, 90, 100 °C) during 60 min and then immediately cooled in a water bath. Samples of *Ulva* from each temperature treatment were withdrawn at 5, 15, 30 and 60 min, and then preincubated with a solution of bovine trypsin (1 µg mL^−1^. T8003 from Sigma Aldrich, SL. Saint Louis, MO, USA) during 60 min at room temperature at a ratio of 500 µg of *Ulva* per µg trypsin. After that, trypsin activity was assayed according to Erlanger et al. [42] using BAPNA (Nα-Benzoyl-DL-arginine 4-nitroanilide hydrochloride) as substrate. Enzyme inhibition was expressed as the percentage of trypsin inhibition after comparing with a control assay carried out without *Ulva*. SBTI was used as positive control of the inhibition assay.

In addition, the formation of proteinaceous enzyme-inhibitor complexes was determined by using substrate-SDS-PAGE electrophoresis gels. Samples were prepared by preincubating crude or heat-treated (100 °C, 5 min) *Ulva* extracts with a trypsin solution (1 µg mL^−1^) at a ratio of 500 µg of *Ulva* per µg trypsin for 0, 30 and 60 min at room temperature. Samples were mixed (1:1) with SDS sample buffer (0.125 M Tris HCl, pH 6.8; 4% (*w*/*v*) SDS; 10% (*v*/*v*) β-mercaptoethanol; 20% (*v*/*v*) glycerol; 0.04% (*w*/*v*) bromophenol blue and SDS-PAGE was performed according to Laemmli [39] using 12% polyacrylamide gels (100 V per gel, 45 min, 4 °C). After electrophoresis, gels were washed with distilled water prior to staining with Coomassie Brilliant Blue R-250 in a methanol-acetic acid solution overnight. Finally, distaining was done with a methanol-acetic acid-water solution. In addition, 5 μL of a wide-range molecular weight marker (S-84445 SigmaMarker™, St. Louis, MO, USA) were included in each gel. The molecular marker consisted of 12 proteins ranging from 6.5 kDa (aprotinin, bovine lung) to 200 kDa (myosin, porcine heart).

#### 4.5.2. Trypsin and Chymotrypsin Inhibition Kinetics

Inhibition kinetics were conducted according to Bijina et al. [30], with minor modifications, using trypsin and chymotrypsin from bovine pancreas (T8003 and C4129 from Sigma Aldrich, SL) and different concentrations of the synthetic substrate. An aliquot of 10 µL of each protease (1 mg mL^−1^) was pre-incubated with different concentrations of *Ulva* (from 0 to 500 µg *Ulva* per µg trypsin) for 60 min. Later on, trypsin and chymotrypsin activities of the pre-incubated mixtures were assayed using various concentrations of BAPNA (Nα-Benzoyl-DL-arginine 4-nitroanilide hydrochloride) (from 0.10 to 0.75 mM) according to Erlanger et al. [42], or SAPNA (N-succinyl-(Ala)2-Pro-Phe-P-nitroanilide) (from 0.05 to 0.5 mM) according to DelMar et al. [43], respectively, in 50mM Tris-HCl, 10mM CaCl_2_ buffer, pH 8.5. 

The activity of the enzymatic reaction (v) based on the rate of change in absorbance (405 nm) of the reaction mixture was determined for each substrate concentration [S] assayed. Lineweaver–Burk curves, 1/v versus 1/[S], were plotted and the Michaelis constant (K*m*) and the maximum rate of reaction (V_max_) were calculated for classifying the pattern of inhibition generated by the *Ulva* extract (competitive, uncompetitive or non-competitive).

### 4.6. Statistical Analysis

The results were expressed as mean ± standard deviation. In order to test data normality and variance homogeneity, the Kolmogorov–Smirnov test and Levene’s F-test were used, respectively. Data with parametric distribution were analyzed using one-way analysis of variance (ANOVA), and the significant differences between treatments (*p* < 0.05) were determined using Tukey’s multiple comparison test. Data with nonparametric distribution were analyzed by using Kruskal–Wallis test, and significant differences were determined using box-and-whisker plot graphs. All statistical analyses were performed using the Statgraphics Plus 4.0 (Rockville, MD, USA) software.

## 5. Conclusions

This work showed the presence of protease inhibitors in *Ulva* able to inhibit digestive proteases of commercial fish species. The inhibitory capacity was dose-dependent. From a physiological point of view, high dietary inclusion of crude *Ulva* would be requested to achieve higher inhibition values. The thermal treatment during feed processing is high enough to inactivate the inhibitors from *U. ohnoi*; hence, it can be used efficiently as potential sustainable ingredient for aquafeeds.

## Figures and Tables

**Table 1:** The values of the estimated coefficient of protein degradation (CPD) and total amino acids released measured after 90 min of in vitro hydrolysis of casein by fish digestive proteases in the presence of different concentrations (0, 500, 1000 and 1500 µg *Ulva* per UA) of crude (CR-*Ulva*) and heat-treated (HT-*Ulva*) *Ulva ohnoi*.

Fish vs. *Ulva* Concentration	CPD (%)	Total Amino Acids Released (g 100 g protein^−1^)
***Sparus aurata***		
Control	91.6 ± 2.3 ^d^	22.1 ± 2.3 ^d^
CR-*Ulva* 500	77.9 ± 2.0 ^c^	15.4 ± 0.1 ^c^
CR-*Ulva* 1000	62.6 ± 4.1 ^b^	11.1 ± 0.7 ^b^
CR-*Ulva* 1500	45.9 ± 2.0 ^a^	7.4 ± 2.4 ^a^
HT-*Ulva* 500	91.4 ± 2.1 ^d^	20.7 ± 1.0 ^d^
HT-*Ulva* 1000	90.6 ± 1.0 ^d^	20.8 ± 1.6 ^d^
HT-*Ulva* 1500	89.2 ± 1.7 ^d^	20.7 ± 2.7 ^d^
*p*	<0.001	<0.001
***Solea senegalensis***		
Control	91.9 ± 2.2 ^d^	22.1 ± 1.5 ^c^
CR*-Ulva* 500	84.3 ± 0.4 ^c^	19.6 ± 0.8 ^c^
CR*-Ulva* 1000	66.1 ± 1.5 ^b^	12.8 ± 0.3 ^b^
CR*-Ulva* 1500	41.8 ± 4.4 ^a^	8.5 ± 1.0 ^a^
HT*-Ulva* 500	89.9 ± 1.2 ^d^	21.9 ± 1.8 ^c^
HT*-Ulva* 1000	89.1 ± 2.1 ^d^	21.5 ± 2.0 ^c^
HT*-Ulva* 1500	88.2 ± 1.3 ^d^	20.8 ± 0.8 ^c^
*p*	<0.001	<0.001
***Dicentrarchus labrax***		
Control	96.2 ± 1.2 ^c^	22.3 ± 2.5 ^d^
CR-*Ulva* 500	91.2 ± 0.8 ^b^	16.3 ± 1.0 ^c^
CR-*Ulva* 1000	69.9 ± 1.4 ^a^	11.8 ± 0.3 ^b^
CR-*Ulva* 1500	66.6 ± 2.5 ^a^	8.2 ± 0.3 ^a^
HT-*Ulva* 500	95.1 ± 1.8 ^b,c^	22.0 ± 2.3 ^d^
HT-*Ulva* 1000	94.1 ± 3.2 ^b,c^	21.3 ± 0.6 ^d^
HT-*Ulva* 1500	93.4 ± 1.4 ^b^	20.1 ± 0.6 ^d^
*p*	<0.001	<0.001

The values are mean ± SD of triplicate determinations. Within each fish species, the values in the same column with different lowercase letters indicate significant differences (*p* < 0.05) owing to *Ulva* extract. ^a,b,c,d^ the superscripts indicate the treatments with significant differences.

**Table 2:** Kinetic analysis of trypsin and chymotrypsin enzymes in the presence of *Ulva*.

*Ulva* Concentration	Trypsin		Chymotrypsin	
	K_m_ (mM)	V_max_ (µMmol pNA min^−1^)	K_m_ (mM)	V_max_ (µMmol pNA min^−1^)
Without *Ulva*	0.65	7.03	0.15	5.92
200 µg *Ulva* per µg enzyme	0.73	5.54	0.20	5.57
300 µg *Ulva* per µg enzyme	0.87	4.71	0.21	3.15
400 µg *Ulva* per µg enzyme	1.58	4.43	0.23	2.38
500 µg *Ulva* per µg enzyme	2.53	4.12	0.25	1.65

K_m_: Michaelis constant; V_max_: maximum rate of reaction; pNA: para-nitroaniline.

## References

[1] Prabu E., Rajagopalsamy C.B.T., Ahilan B., Santhakumar R., Jeevagan I.J.M.A., Renuhadevi M. (2017). An overview of anti-nutritional factors in fish feed ingredients and their effects in fish. J. Aquac. Trop..

[2] Thakur A., Sharma V., Thakur A. (2019). An overview of anti-nutritional factors in food. Int. J. Chem. Stud..

[3] Glencross B.D., Baily J., Berntssen M.H., Hardy R., MacKenzie S., Tocher D.R. (2019). Risk assessment of the use of alternative animal and plant raw material resources in aquaculture feeds. Rev. Aquac..

[4] Chakraborty P., Mallik A., Sarang N., Lingam S.S. (2019). A review on alternative plant protein sources available for future sustainable aqua feed production. Int. J. Chem. Stud..

[5] Hajra A., Mazumder A., Verma A., Ganguly D.P., Mohanty B.P., Sharma A.P. (2013). Antinutritional factors in plant origin fish feed ingredients: The problems and probable remedies. Adv. Fish Res..

[6] Mæhre H.K. (2015). Seaweed Proteins—How to Get to Them? Effects of Processing on Nutritional Value, Bioaccessibility and Extractability. Ph.D. Thesis.

[7] Diken G., Demir O., Naz M. (2016). The inhibitory effects of different diets on the protease activities of *Argyrosomus regius* (Pisces, Scianidae) larvae as a potential candidate species. J. Appl. Anim. Res..

[8] Vizcaíno A.J., Fumanal M., Sáez M.I., Martínez T.F., Moriñigo M.A., Fernández-Díaz C., Anguís V., Balebona M.C., Alarcón F.J. (2019). Evaluation of *Ulva ohnoi* as functional dietary ingredient in juvenile Senegalese sole (*Solea senegalensis*): Effects on the structure and functionality of the intestinal mucosa. Algal Res..

[9] Guerreiro I., Magalhães R., Coutinho F., Couto A., Sousa S., Delerue-Matos C., Domingues V.F., Oliva-Teles A., Peres H. (2019). Evaluation of the seaweeds *Chondrus crispus* and *Ulva lactuca* as functional ingredients in gilthead seabream (*Sparus aurata*). J. Appl. Phycol..

[10] Pereira V., Marques A., Gaivão I., Rego A., Abreu H., Pereira R., Santos M.A., Guilherme S., Pacheco M. (2019). Marine macroalgae as a dietary source of genoprotection in gilthead seabream (*Sparus aurata*) against endogenous and exogenous challenges. Comp. Biochem. Phys. C.

[11] Tapia-Paniagua S.T., Fumanal M., Anguis V., Fernandez-Diaz C., Alarcón F.J., Moriñigo M.A., Balebona M.C. (2019). Modulation of intestinal microbiota in *Solea senegalensis* fed low dietary level of *Ulva ohnoi*. Front. Microbiol..

[12] Peixoto M.J., Magnoni L., Gonçalves J.F., Twijnstra R.H., Kijjoa A., Pereira R., Palstra A.P., Ozório R.O. (2019). Effects of dietary supplementation of *Gracilaria* sp. extracts on fillet quality, oxidative stress, and immune responses in European seabass (*Dicentrarchus labrax*). J. Appl. Phycol..

[13] Silva D.M., Valente L.M.P., Sousa-Pinto I., Pereira R., Pires M.A., Seixas F., Rema P. (2015). Evaluation of IMTA-produced seaweeds (*Gracilaria, Porphyra*, and *Ulva*) as dietary ingredients in Nile tilapia, *Oreochromis niloticus* L., juveniles. Effects on growth performance and gut histology. J. Appl. Phycol..

[14] Oliveira M.N., Ponte-Freitas A.L., Urano-Carvalho A.F., Taveres-Sampaio T.M., Farias D.F., Alves-Teixera D.I., Gouveia S.T., Gomes-Pereira J., Castro-Catanho de Sena M.M. (2009). Nutritive and non-nutritive attributes of washed-up seaweeds from the coast of Ceará. Food Chem..

[15] Francis G., Makkar H.P., Becker K. (2001). Antinutritional factors present in plant-derived alternate fish feed ingredients and their effects in fish. Aquaculture.

[16] Le Bourvellec C., Renard C.M. (2012). Interactions between polyphenols and macromolecules: Quantification methods and mechanisms. Crit. Rev. Food Sci. Nutr..

[17] Sáez M.I., Martinez T.F., Alarcón F.J. (2013). Effect of the dietary of seaweeds on intestinal proteolytic activity of juvenile sea bream *Sparus Aurata*. Int. Aquafeed.

[18] Deivasigamani B., Subamanian V. (2016). Applications of immunostimulants in aquaculture: A review. Int. J. Curr. Microbiol. App. Sci..

[19] Moutinho S., Linares F., Rodriguez J.L., Sousa V., Valente L.M.P. (2018). Inclusion of 100% seaweed meal in diets for juvenile and on-growing life stages of Senegaleses sole (*Solea senegalensis*). J. Appl. Phycol..

[20] Haard N.F., Dimes L.E., Arndt R.E., Dong F.M. (1996). Estimation of protein digestibility. IV. Digestive proteinases from the pyloric caeca of coho salmo (*Oncorhynchus kisutch*) fed diets containing soybean meal. Comp. Biochem. Physiol..

[21] Krogdahl Å., Penn M., Thorsen J., Refstie S., Bakke A.M. (2010). Important antinutrients in plant feedstuffs for aquaculture: An update on recent findings regarding responses in salmonids. Aquac. Res..

[22] Magnoni L.J., Martos-Sitcha J.A., Queiroz A., Calduch-Giner J.A., Gonçalves J.F.M., Rocha C.M.R., Abreu H.T., Schrama J.W., Ozorio R.O.A., Pérez-Sánchez J. (2017). Dietary supplementation of heat-treated *Gracilaria* and *Ulva* seaweeds enhanced acute hypoxia tolerance in gilthead sea bream (*Sparus aurata*). Biol. Open.

[23] Bandara T. (2018). Alternative feed ingredients in aquaculture: Opportunities and challenges. J. Entomol. Zool. Stud..

[24] Vizcaíno A.J., Sáez M.I., Martínez T.F., Acién F.G., Alarcón F.J. (2019). Differential hydrolysis of proteins of four microalgae by the digestive enzymes of gilthead sea bream and Senegalese sole. Algal Res..

[25] He H., Li X., Kong X., Hua Y., Chen Y. (2017). Heat-induced inactivation mechanism of soybean Bowman-Birk inhibitors. Food Chem.

[26] Vagadia B.H., Vanga S.K., Raghavan V. (2017). Inactivation methods of soybean trypsin inhibitor—A review. Trends Food Sci. Technol..

[27] Maehre H.K., Edvinsen G.K., Eilertsen K.E., Elvevoll E.O. (2016). Heat treatment increases the protein bioaccessibility in the red seaweed dulse (*Palmaria palmata*), but not in the brown seaweed winged kelp (*Alaria esculenta*). J. Appl. Phycol..

[28] Bijina B., Chellappan S., Krishna J.G., Basheer S.M., Elyas K.K., Bahkali A.H., Chandrasekaran M. (2011). Protease inhibitor from *Moringa oleifera* with potential for use as therapeutic drug and as seafood preservative. Saudi J. Biol. Sci..

[29] Chang T.S. (2009). An updated review of tyrosinase inhibitors. Int. J. Mol. Sci..

[30] Sharma R. (2012). Enzyme inhibition: Mechanisms and scope. Enzyme inhibition and bioapplications.

[31] Dantzger M., Vasconcelos I.M., Scorsato V., Aparicio R., Marangoni S., Macedo M.L.R. (2015). Bowman—Birk proteinase inhibitor from *Clitoria fairchildiana* seeds: Isolation, biochemical properties and insecticidal potential. Phytochemistry.

[32] Macedo M.L.R., Freire M.G.M., Cabrini E.C., Toyama M.H., Novello J.C., Marangoni S. (2003). A trypsin inhibitor from *Peltophorum dubium* seeds active against pest proteases and its effect on the survival of *Anagasta kuehniella* (Lepidoptera:Pyralidae). Biochim. Biophys. Gen. Subj..

[33] Xu X., Liu J., Wang Y., Si Y., Wang X., Wang Z., Zhang Q., Yu H., Wang X. (2018). Kunitz-type serine protease inhibitor is a novel participator in anti-bacterial and anti-inflammatory responses in Japanese flounder (*Paralichthys olivaceus*). Fish Shellfish Immunol..

[34] Harry J.B., Steiner R.F. (1970). A soybean proteinase inhibitor: Thermodynamic and kinetic parameters of association with enzymes. Eur. J. Biochem..

[35] Avilés-Gaxiola S., Chuck-Hernández C., del Refugio Rocha-Pizaña M., García-Lara S., López-Castillo L.M., Serna-Saldívar S.O. (2018). Effect of thermal processing and reducing agents on trypsin inhibitor activity and functional properties of soybean and chickpea protein concentrates. LWT Food Sci. Technol..

[36] Bradford M. (1976). A rapid and sensitive method for the quantitation of microgramquantities of protein utilizing the principle of protein-dye binding. Anal. Biochem..

[37] Alarcón F.J., Díaz M., Moyano F.J., Abellán E. (1998). Characterization and functional properties of digestive proteases in two sparids; gilthead sea bream (*Sparus aurata*) and common dentex (*Dentex dentex*). Fish Physiol. Biochem..

[38] Alarcón F.J., García-Carreño F.L., Navarrete del Toro M.A. (2001). Effect of plant protease inhibitors on digestive proteases in two fish species, *Lutjanus argentiventris* and *L. novemfasciatus*. Fish Physiol. Biochem..

[39] Laemmli U.K. (1970). Cleavage of structural proteins during the assembly of the head of bacteriophage T4. Nature.

[40] Hamdan M., Moyano F.J., Schuhardt D. (2009). Optimization of a gastrointestinal model applicable to the evaluation of bioaccessibility in fish feeds. J. Sci. Food Agric..

[41] Church F.C., Swaisgood H.E., Porter D.H., Catignani G. (1983). Spectrophotometric assay using o-phthaldehyde for determination of proteolysis in milk proteins. J. Dairy Sci..

[42] Erlanger B., Kokowsky N., Cohen W. (1961). The preparation and properties of two new chromogenic substrates of trypsin. Arch. Biochem. Biophys.

[43] DelMar E.G., Largman C., Brodrick J.W., Geokas M.C. (1979). A sensitive new substrate for chymotrypsin. Anal. Biochem..

